# A rare pancreatic tumor unamenable to accurate diagnosis by EUS–guided tissue acquisition

**DOI:** 10.1097/eus.0000000000000135

**Published:** 2025-11-03

**Authors:** Naho Kondo, Akihiko Kida, Jun Asai, Tatsuya Yamashita, Takeshi Urabe, Taro Yamashita

**Affiliations:** 1 Department of Gastroenterology, Public Central Hospital of Matto Ishikawa, Hakusan, Japan; 2 Department of Gastroenterology, Kanazawa University Hospital, Kanazawa, Japan; 3 Department of Gastroenterology, Saiseikai Kanazawa Hospital, Kanazawa, Japan.

An 85-year-old woman was diagnosed with an 11-mm nodular lesion in the pancreatic body, with mild dilation of the main pancreatic duct (MPD) in the pancreatic tail on computed tomography [Figure 1]. Blood tests showed normal CEA and CA19–9 levels. Magnetic resonance cholangiopancreatography revealed 3.5-mm MPD dilation in the pancreatic tail [Figure 2]. Fluorodeoxyglucose-positron emission tomography demonstrated mild accumulation at the nodular lesion. Because of the diagnostic challenge in distinguishing pancreatic cancer from other pancreatic diseases, EUS–guided tissue acquisition (EUS-TA) was planned. EUS revealed an 11-mm hypoechoic nodular lesion in the pancreatic body [Figure 3]. EUS-TA was performed using a 22-gauge Franseen needle, and pancreatic cancer was strongly suspected [Figures 4 and 5]. We performed distal pancreatectomy. Histological examination revealed infiltration of lymphocytes and plasma cells in the stroma surrounding low-grade pancreatic intraepithelial neoplasia (PanIN) in the pancreatic body [Figure 6]. Notably, no pancreatic cancer was observed. Immunohistochemistry revealed over 100 IgG4-positive plasma cells per high magnification field in the stroma surrounding PanIN [Figure 7]. In the tail region, no infiltration of lymphocytes or plasma cells was observed, and no PanIN was present. The postoperative diagnosis was coexistence of low-grade PanIN and focal type 1 autoimmune pancreatitis (AIP).

**Figure 1:**
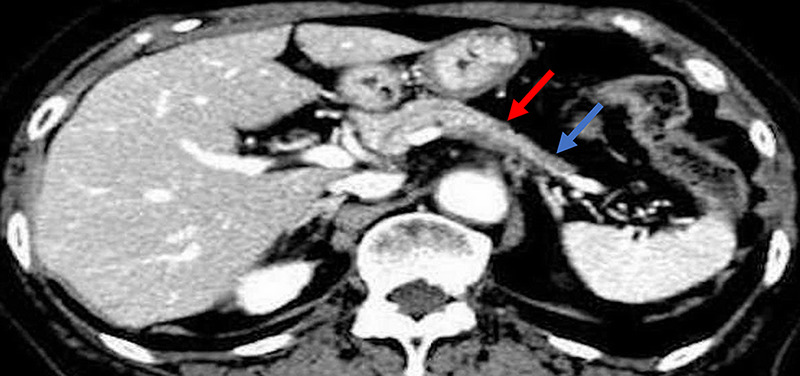
Contrast-enhanced computed tomography showed an 11-mm nodular lesion (red arrow) in the pancreatic body, which appeared hypodense in the early phase. Furthermore, mild dilation of the main pancreatic duct and atrophy of the pancreatic tail on the caudal side of the nodular lesion were observed. The blue arrow showed mild dilation of the main pancreatic duct and atrophy of the pancreatic tail.

**Figure 2:**
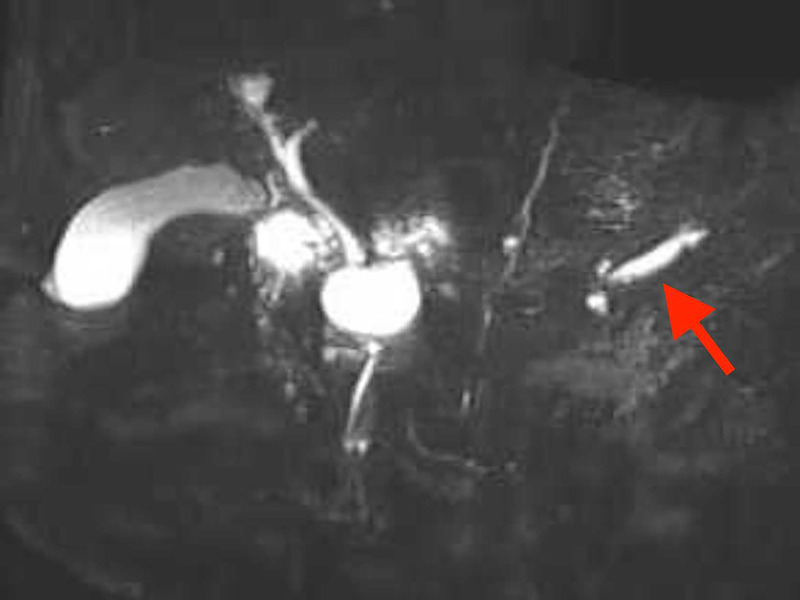
Magnetic resonance cholangiopancreatography showed a 3.5-mm dilation (red arrow) of the main pancreatic duct in the pancreatic tail.

**Figure 3:**
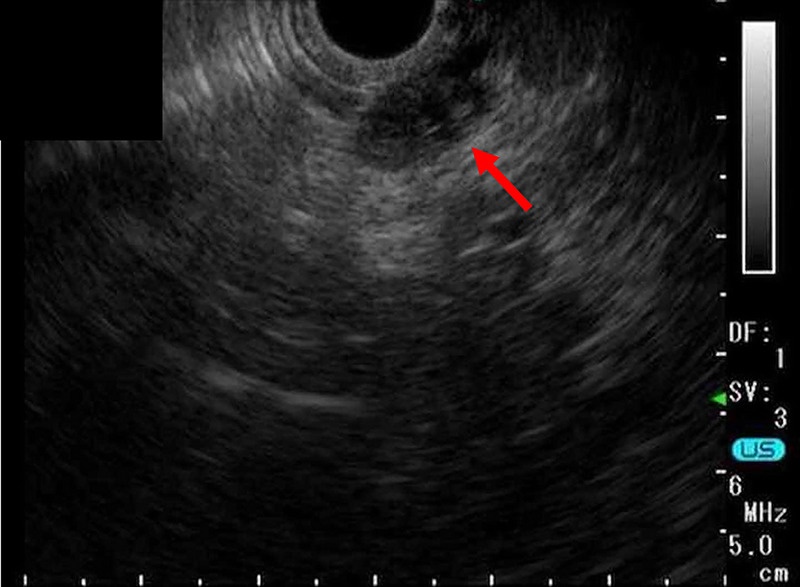
EUS revealed an 11-mm hypoechoic nodular lesion (red arrow) in the pancreatic body.

**Figure 4:**
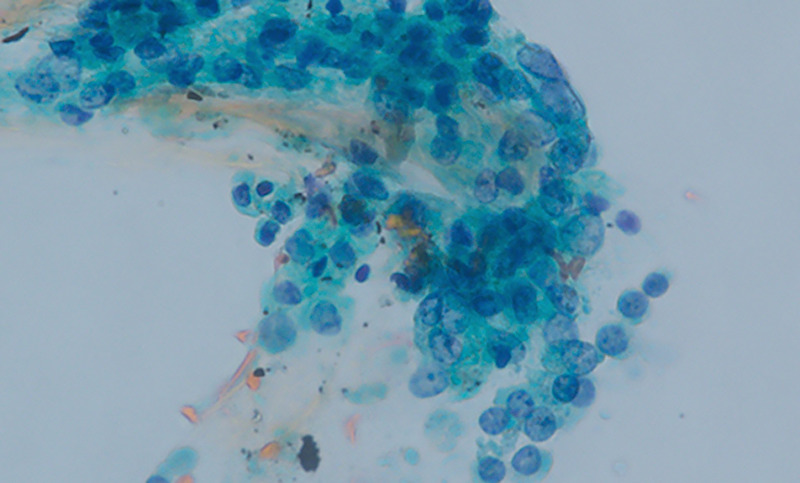
Cytological examination of the EUS–guided tissue acquisition sample with Papanicolaou staining showed irregular nuclear morphology and nuclear arrangement of the glandular cells, which was suspicious of adenocarcinoma (magnification ×100).

**Figure 5:**
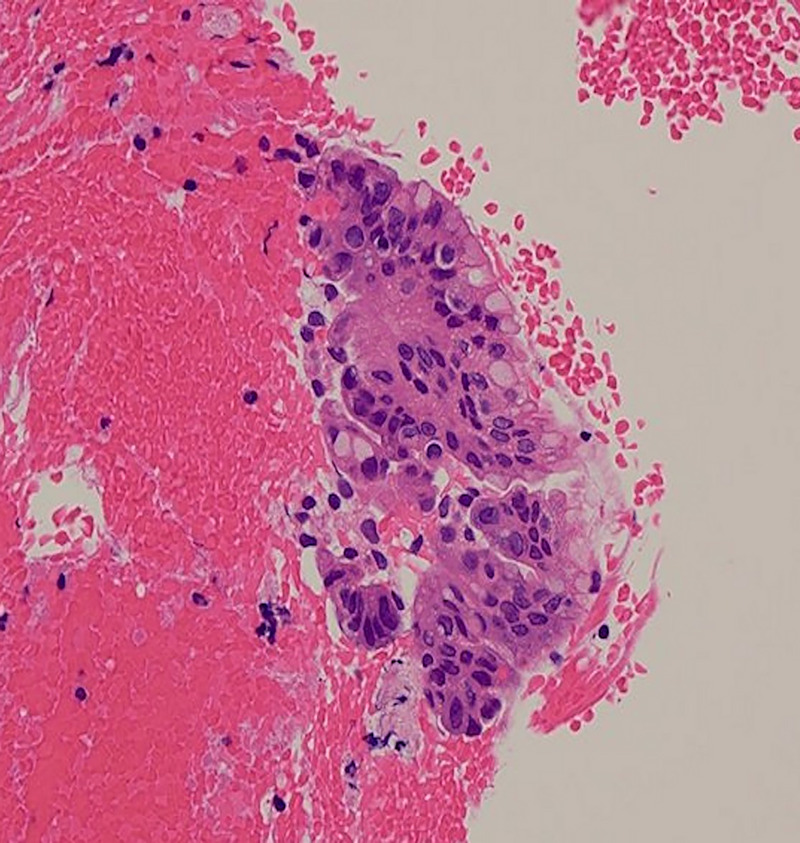
Histological examination of the EUS–guided tissue acquisition sample with hematoxylin and eosin staining showed atypical epithelium suggestive of adenocarcinoma (magnification ×40).

**Figure 6:**
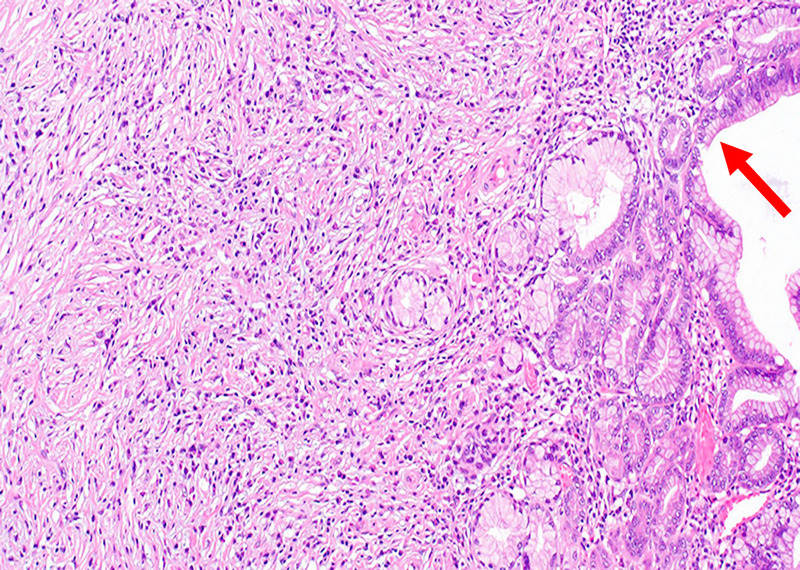
Histological examination of the surgical specimen with hematoxylin and eosin staining showed infiltration of numerous lymphocytes and plasma cells in the stroma around low-grade pancreatic intraepithelial neoplasia (red arrow) in the pancreatic body (magnification ×20).

**Figure 7:**
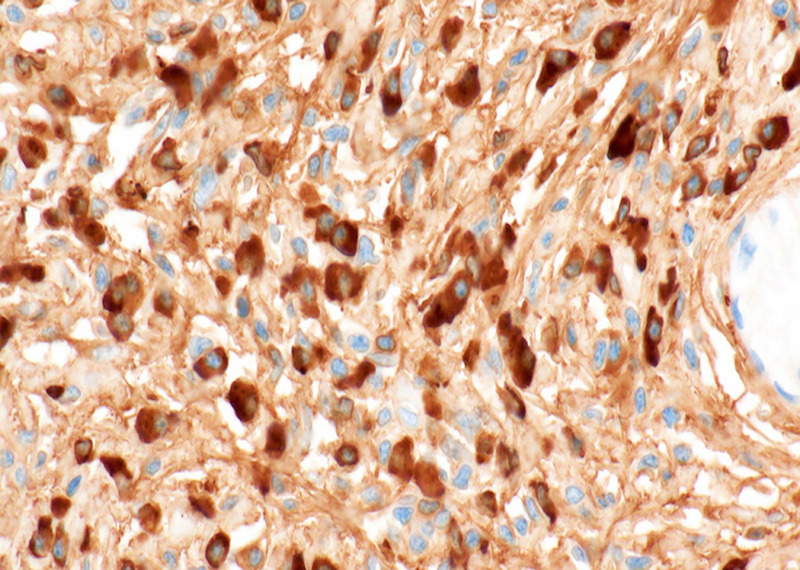
Immunohistochemistry examination showed over 100 IgG4-positive plasma cells per high-magnification field in the stroma surrounding low-grade pancreatic intraepithelial neoplasia (magnification ×100). The postoperative diagnosis was coexistence of low-grade pancreatic intraepithelial neoplasia and focal type 1 autoimmune pancreatitis in the pancreatic body.

Although PanIN has been reported to increase with age and is found in 33% of men older than 60 years,^[1]^ it is unlikely that the coexistence of PanIN and AIP in our case was incidental. As PanIN lesions were observed only in areas of AIP, AIP may have contributed to the development of PanIN. Few studies have reported that AIP is associated with the development of PanIN, and whether AIP is a risk factor for the development of pancreatic cancer is controversial.^[2]^ Our case is valuable because it may suggest a process in which AIP is involved in the development of neoplastic lesions such as PanIN. Furthermore, although it is well known that EUS-TA has higher diagnostic yields for pancreatic solid tumors,^[3]^ it should be noted that unusual pancreatic solid tumor such as the coexistence of PanIN and AIP may be difficult to accurately diagnose even with EUS-TA.

## Acknowledgments

None.

## Source of Funding

None.

## Ethical Approval

This case was conducted in accordance with the ethical standards described in the latest revision of the Declaration of Helsinki.

## Informed Consent

Informed consent for patient participation and publication was received from the patient.

## Conflicts of Interest

All the authors declare that no conflicts of interest exist.

## Author Contributions

A. Kida did the concept and design. N. Kondo and A. Kida did the data acquisition. N. Kondo and A. Kida contributed to the analysis and interpretation of data. N. Kondo, A. Kida, J. Asai, T. Yamashita, T. Urabe, and T. Yamashita did the manuscript writing and review. T. Urabe and T. Yamashita supervised the study.

## Data Availability Statements

All data relevant to the case are included in the article.
